# Spinal pain increases the risk of becoming overweight in Danish schoolchildren

**DOI:** 10.1038/s41598-021-89595-5

**Published:** 2021-05-13

**Authors:** Lise Hestbaek, Ellen Aartun, Pierre Côté, Jan Hartvigsen

**Affiliations:** 1 The Chiropractic Knowledge Hub, Odense, Denmark; 2 Department of Sports Science and Clinical Biomechanics, University of Southern Denmark, Odense, Denmark; 3 University of Oslo, Oslo, Norway; 4 Centre for Disability Prevention and Rehabilitation, Ontario Tech University, Toronto, Canada

**Keywords:** Health care, Risk factors

## Abstract

Spinal pain is common in adolescence, and overweight in children and adolescence is an increasing public health problem globally. Since musculoskeletal pain is a known barrier for physical activity which potentially can lead to overweight, the primary objective of this study was to determine if self-reported lifetime spinal pain in 2010 was associated with being overweight or obese in 2012 in a cohort of 1080 normal-weighted Danish children, aged 11–13 years at baseline. Overweight was based on body mass index measured by trained staff. Spinal pain was self-reported by questionnaires during school hours. Estimates were adjusted for relevant covariates. The 2-year incidence rate of overweight was 5.3% (95% CI 3.98–7.58) for children with spinal pain at baseline versus 1.6% (95% CI 0.19–5.45) for children without. There was stepwise and statistically significant increased risk of overweight with increasing frequency of pain and for having pain in more than one part of the spine. Despite the short follow-up time where only 40 children developed overweight, these results indicate that spinal pain might increase the risk of subsequent overweight.

## Introduction

Overweight and obesity in childhood and adolescence has increased substantially over the past four decades with more than 20% of children and adolescents in high-income countries and around 13% in low- and middle-income countries being either overweight or obese in 2013^1^. In 2017, the high prevalence of overweight children was considered as a global health crisis by the WHO, and if current trends continue, it is estimated that more children will be obese than underweight by 2022^2^. Being overweight and obese during adolescence has been correlated with adult coronary heart disease, diabetes and a range of cancers^3^ as well as with premature death^4,5^. Importantly, overweight in childhood is a strong risk factor for overweight in adulthood^6^.

Understanding causal pathways leading to overweight is necessary to develop effective preventive strategies. Body weight, and ultimately obesity, is determined by the interaction between genetic, environmental and psychosocial factors acting through several physiological mediators of food intake and energy expenditure that affect fat deposition^7^. The search for major gene effects in obesity has resulted in the successful identification of some monogenic forms of obesity^8^. However, obesity generally is the result of interactions between genetic susceptibility and diverse lifestyle factors^9^ such as diet and physical activity^10^. These factors are determined through complex interactions between biology and environment. For example, eating behaviors are shaped not only by genetic and other biological factors but also by opportunity to eat, food availability and affordability. Physical activity, another behavioral component, is also conditioned by socioeconomic and cultural factors such as transportation type, videogame play, computer use and occupation. A conjoint secular trend in sedentary lifestyles coupled with increases in energy intake can to a large degree explain the observed increases in mean BMI^7^.

Low back pain is now the leading cause of years lived with disability worldwide, and for 10–14-year-old children it is ranked as number nine^11^. The prevalence of low back pain raises rapidly during adolescence to reach adult level around the age of 18^12^, and the prevalence of non-trivial spinal pain, i.e. pain in the neck, mid back, and low back pain, in 11–14-year-old Danes has been estimated to be around 12%^13^. Spinal pain is associated with reduced physical activity and sports participation in children, for example 25% of Danish children reporting low back pain were less physically active than their peers without pain^14^ and in a clinical sample, 77% reported much or some limitations of activities^15^. In adults, Lake et al. showed that low back pain at age 23 is a risk factor for gaining weight 10 years later^16^. Whether such an association is present earlier in life is unknown, but Paulis et al. reported a cross-sectional association between overweight and low back pain in children and called for cohort studies that explores the association between the two^17^.

The primary objective of this study is to determine if self-reported lifetime spinal pain is associated with being overweight or obese 2 years later in a cohort of normal-weighted Danish children, aged 11–13 years. Secondary objectives are to explore how the frequency of spinal pain episodes and the number of spinal pain sites influence the risk of becoming overweight or obese.

## Methods

### Design

Prospective school-based cohort study with 2 years follow-up.

### Setting

We used data from a cluster-randomized controlled trial, the School site, Play Spot, Active transport, Club fitness and Environment (SPACE) study, to form a cohort of 11–13-year-old normal-weight Danish students. The SPACE study was designed to develop, document, and assess a comprehensive intervention in 14 local school districts in the Region of Southern Denmark that promoted everyday physical activity among 11–13-year-old adolescents with 2 years follow-up. In the randomized trial, no effect of the intervention was detected on physical activity, physical fitness or measures of overweight between the intervention group and control group^18,19^.

### Data collection

We collected baseline data from April to June 2010 and follow-up data from April to June 2012 while the children were at school. In the schools, trained investigators collected anthropometrical data at baseline and follow-up. Participants completed an e-survey, both at baseline and at follow-up 2 years later, that included questions about spinal pain, sports participation, dieting and psychological factors. At follow-up, data about the social class was also included. The teachers observed participants during completion of the questionnaires to avoid interaction between the children. During the same week, the children were also asked to wear an accelerometer, Actigraph GT3X Triaxial Activity Monitor (http://www.theactigraph.com/products/gt3x/) to objectively measure their level of physical activity.

### Participants

The study included all fifth and sixth grade students (aged 11–13, N = 1348) from 14 public schools in the Region of Southern Denmark in 2010. The population at risk included normal-weight and underweight participants only. We calculated the Body Mass Index (BMI) as weight divided by the height squared (kg/m^2^) and used the International Obesity Task Force system to define overweight using age- and gender-specific cut-off points for overweight, relative to 25 kg/m^2^ for adults^20^.

### Exposures

The e-survey contained parts of the Young Spine Questionnaire, that include identical questions for the neck, mid back and low back, i.e. "Have you ever had pain in your neck/mid-back/low back?" with response options "often"/"sometimes"/"once or twice"/"never"^21^. A diagram showed three clearly shaded areas referring to the neck, mid back and low back; these areas were labelled alongside the questions. We used this data to define three exposures:

- Lifetime prevalence of spinal pain. We defined a positive response as a report of “often”, “sometimes” or “once or twice” in any spine site. A negative response is defined as a report of “never” in all three spine sites.
- Frequency of spinal pain. We defined frequency of spinal pain as "often", "sometimes", "once or twice", or "never" in any of the three spine sites. If the frequency of pain differed in the three sites, we used the site with the highest frequency. A missing value in any site resulted in a missing value for this variable.
- Number of spinal pain sites. We summarized the number of positive responses to lifetime prevalence of pain in the three spine sites, resulting in four categories: “0 pain sites”, “1 pain site”, “2 pain sites” or “3 pain sites”. A missing value in any spine site resulted in a missing value for this variable.

### Outcome

The outcome was overweight or obesity, defined as in the definition of participants^20^. The Childhood obesity Outcome Review by Bryant et al. recommends use of BMI as an overweight measure rather than waist circumference because waist circumference is more subject to measurement error than BMI^22^.

### Potential confounders

#### Sociodemographic variables

Sociodemographic variables included sex, age in years, and social class based on the occupation of the father. The social class was measured at follow-up only and categorized as high, middle, low, or unclassified^23^. We assumed that the occupation of the father remained unchanged from baseline to follow-up.

#### Psychological health

Data on psychological health was collected using questions from the Health Behaviour in School-Aged Children: World Health Organization Collaboration Cross-National Survey^24^. The four items were: “In the past 6 months, how often have you (a) been feeling low; (b) been irritable/in a bad mood; (c) felt nervous; (d) had sleep difficulties”. All four questions had four answer options: almost every day/more than once a week/almost every week/almost every month/seldom or never. It has previously been shown in the same cohort, that the four questions all load onto the same factor and therefore can be summarized into one variable with a combined score, ranging from 0 to 16^25^.

#### Objectively measured physical activity

We used the average physical activity defined as the total number of counts divided by the total wear time of the accelerometer across all days with valid accelerometer data (counts per minute). To ensure valid measures of physical activity, we only included data with at least 10 h assessments per day (wear-time) between 6 am to midnight. We defined non-wear time as no activity measured by the accelerometer for at least 60 consecutive minutes as recommended by Toftager et al. and was excluded from the data^26^. Activity was summarized for every 10 s (epoch length). We required at least 3 days with valid data, which has been shown to give a reliable estimate of physical activity in children aged 7 years^27^.

#### Diet

 Two questions covered the dieting habits in the questionnaire: “How many pieces of fruit do you usually eat each day?” and “How many pieces of vegetables do you usually eat each day?” (0/1/2/3/4/5/6 or more). The recommendations for vegetable and fruit consumption vary across the European Region, but we merged the two variables and dichotomized it into meeting the Danish recommendation (≥ 6 servings per day) or not meeting the Danish recommendation (< 6 servings per day)^28^.

### Statistical analysis

We computed descriptive statistics of the exposure, outcome and potential confounders, reporting numbers and percentages for dichotomized or categorical variables, means with standard deviations for normally distributed data, and median with interquartile range for non-normally distributed data. There were very few missing data and therefore imputation was not performed. However, for transparency and ease of interpretation, descriptive statistics for both the population at risk at baseline and the sample with complete follow-up are reported.

The 2 year incidence rates for the different spinal pain groups were calculated and statistical significance for differences were determined using Chi^2^ tests for the dichotomous variable (SP ever) and for the frequency and pain sites, a nonparametric test for trend across ordered groups developed by Cuzick, which is an extension of the Wilcoxon rank-sum test was used.

We computed the crude association between the spinal pain and overweight using a log-binomial regression to calculate the relative risk (RR) with 95% confidence interval (CI) with the exposure variables as categorical.

**Figure 1:**
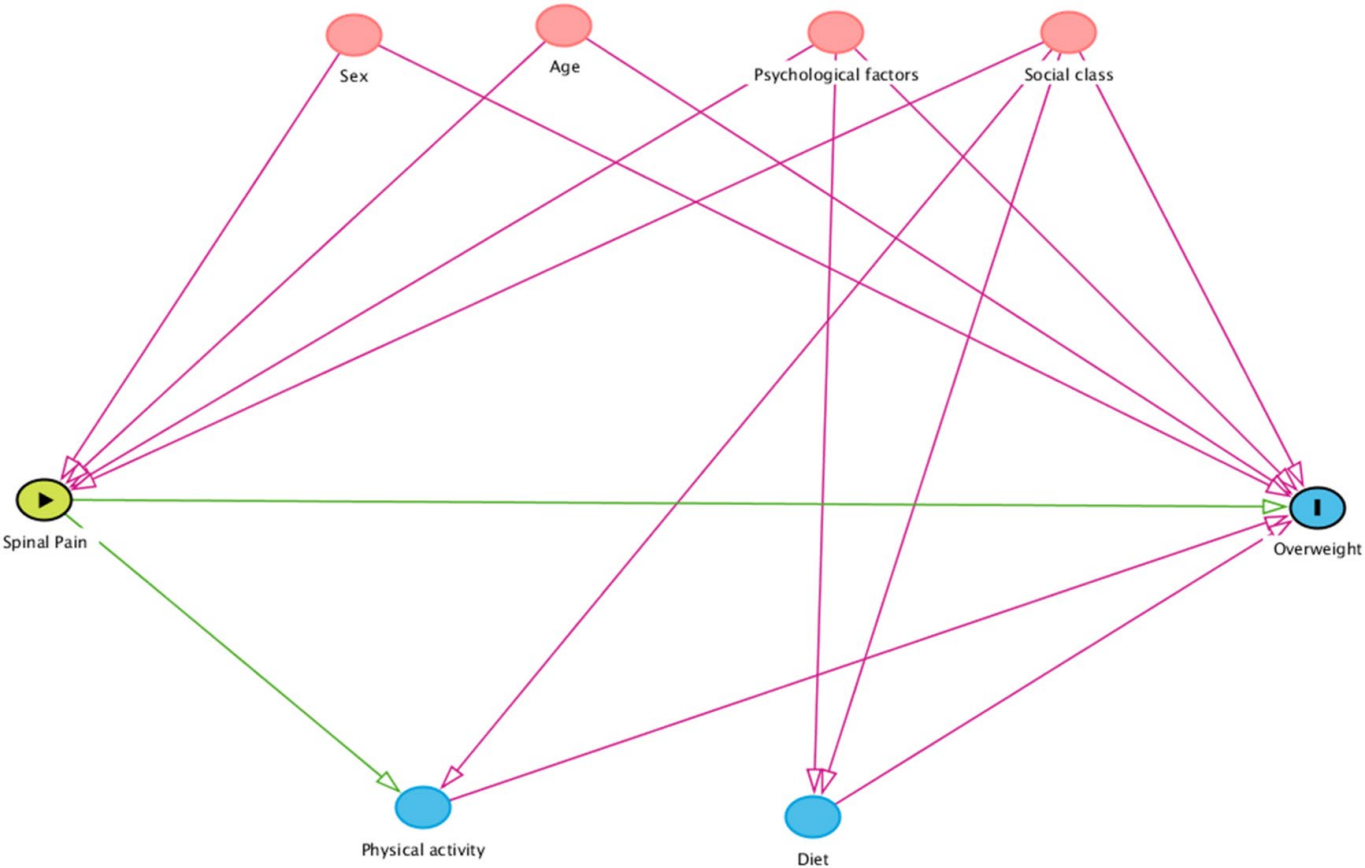
Directed acyclic graph.

We repeated this analysis in multivariable models with inclusion of potential confounders performing both a theory driven and a data-driven approach. In the theory driven approach, we constructed a directed acyclic graph (DAG)^29^. Age and sex are associated with both overweight, with more boys than girls being overweight^2,10^, and with spinal pain being more common in girls^12,13^. As described in the introduction, social factors, psychological health, diet and level of physical activity may all be linked to the development of obesity^7,9,10^. Likewise, evidence suggests that social class and psychological health are associated with spinal pain^13,30^. Despite several investigations into the relationship between level of physical activity and spinal pain, the results are still inconclusive^31^. If decreased physical activity is a potential consequence of spinal pain^14,15^, it is part of the causal pathway between spinal pain and overweight and should therefore *not* be controlled for. Likewise, there is no indication that dietary factors can lead to spinal pain, and therefore diet cannot be considered as a confounder, but might be on the causal pathway between spinal pain and overweight. Based on the assumed relationships between the proposed confounders and spinal pain and overweight, respectively, a directed acyclic graph illustrating the proposed covariate matrix was constructed (Fig. 1). The minimally sufficient adjustment set for estimating the total effect of spinal pain on overweight was sex, age, social class, and psychological health.

To be a true confounder the variable must be related to both predictor and outcome^29^, but for the data-driven model, we chose an approach where variables were included if they were associated with either predictor or outcome, i.e. spinal pain at baseline or overweight at follow-up. The consequent risk of overadjustment will be evaluated. The statistical significance was tested with Chi square tests for binary and categorical variables, and with bivariate regression analyses for the continuous variables. Variables were included if the p-value for the relationship to either predictor or outcome was < 0.10.

We checked the models for multicollinearity, and we did not include variables in the same model if the Spearman correlation coefficient between two variables exceeded 0.4. If individual variance inflation factors (VIF) were below 10 and the mean of VIFs was below three, we considered the model as acceptable.

Although BMI is recommended as the most reliable overweight measurement^22^, waist-to-height ratio has been shown to be more sensitive in predicting lifestyle disorders such as diabetes and cardiovascular diseases^32,33^. Therefore, we performed a sensitivity analysis where all analyses were repeated with overweight based on the waist-to-height ratio. This was calculated as the waist circumference (cm) divided by the height (cm). Participants with a waist-to-height ratio ≥ 0.5 were defined as being overweight^32^. Since we are not aware of any age and sex standardized definition of overweight based on waist-to-height ratio, we adjusted these analyses for sex and age.

All analyses were performed in STATA/IC, version 16. Reporting follows the STROBE checklist.

### Ethics approval

The Danish National Committee on Health Research Ethics reviewed the study protocol and concluded that formal ethical approval was not required. According to Danish law, a study that does not contain invasive tests or interventions aimed at individuals does not require ethics approval. Due to Prof. Côté’s affiliation with the Ontario Tech University, approval was obtained from the Ontario Tech University Research Ethics Board (#14384). The study was registered and listed in the Danish Data Protection Agency (reference number: 2009-41-3628 and 2010-41-5147) and registered in the Current Controlled Trials (ISRCTN79122411).

Personalised information about the SPACE study was distributed to parents and students. Parents of the participating adolescents received a passive informed consent form that explained the nature and procedures of the study. Both adolescents and parents were informed that it was possible to withdraw at any stage of the study.

The study was performed in accordance with the ethical standards as laid down in the 1964 Declaration of Helsinki and its later amendments or comparable ethical standards^34^.

### Consent to participate

Participation did not require parental consent, but the parents could opt-out on behalf of their child at any time. We informed the Regional Ethics Committee for Southern Denmark about the study and data collection.

## Results

**Figure 2:**
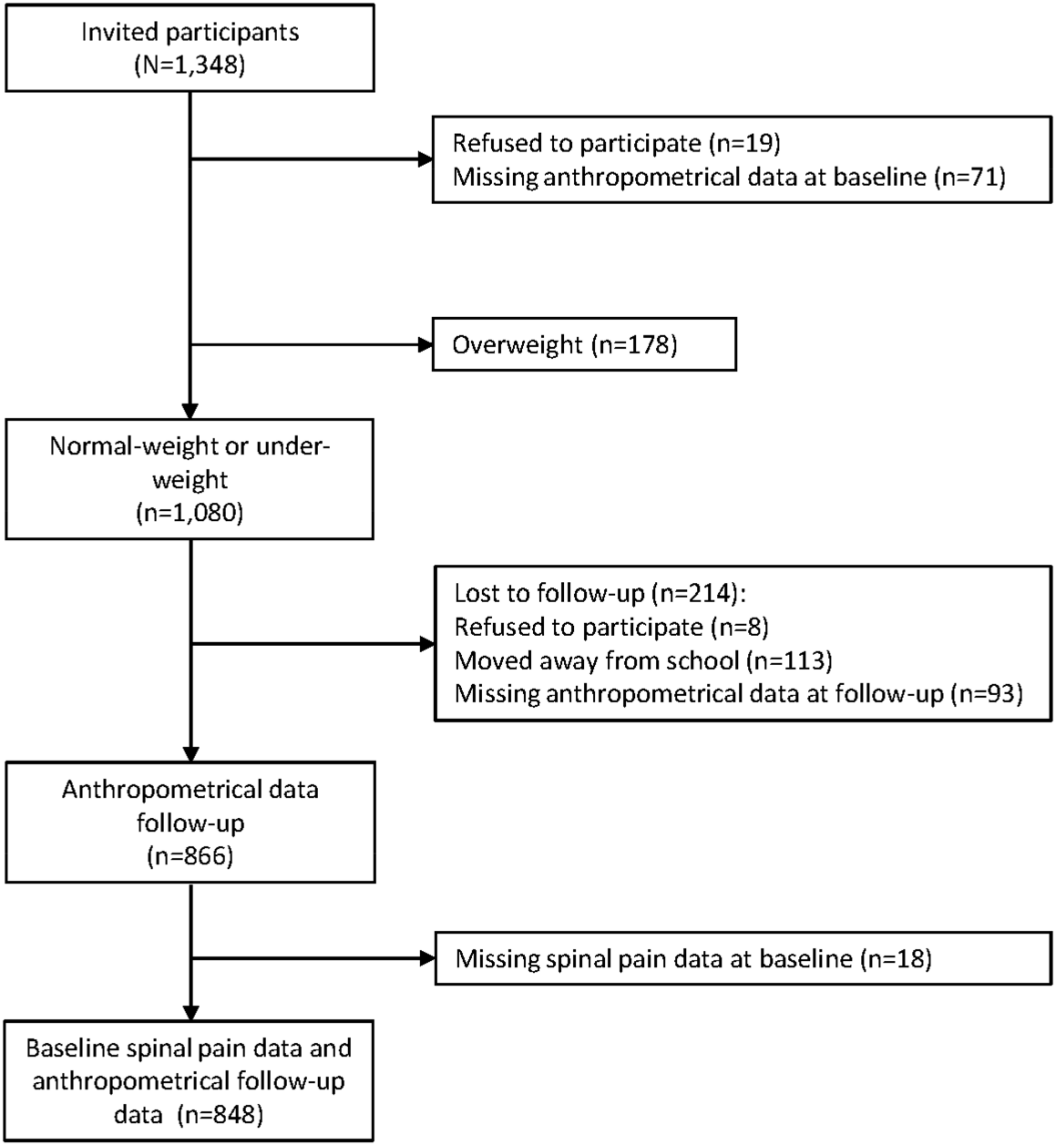
Flowchart of participation.

Of 1348 invited participants, 19 refused to participate, and anthropometric data was missing for 71, resulting in a participation rate of 93% at baseline. Of these, 178 children were classified as being overweight or obese at baseline and excluded from the population at risk, and thus 1080 under- or normal weight children were eligible for our cohort. Anthropometric and spinal pain data were available at follow-up for 848 of these children, resulting in a participation rate of 79% (Fig. 2).

Baseline description of the population at risk at baseline and the sample with complete follow-up, used for analysis, can be seen in Table 1. No significant (neither clinically nor statistically) differences were detected.

**Table 1:** Baseline characteristics of a population-based sample normal-weighted Danish children aged 11–13 years in 2010.

	Eligible (N = 1080) n (%)	Included (N = 848) n (%)
Sex (boys)	554 (51.3)	448 (52.8)
Age, median years (interquartile range)	12.6 (12.1–13.1)	12.6 (12.1–13.3)
Spinal pain ever	902 (85.5)	719 (84.8)
**Frequency of spinal pain**		
Never	153 (14.5)	129 (15.2)
Once or twice	423 (40.1)	337 (39.7)
Sometimes	341 (32.3)	280 (33.0)
Often	138 (13.1)	102 (12.0)
**Number of spinal pain sites**		
0	153 (14.5)	129 (15.2)
1	250 (23.7)	203 (23.9)
2	322 (30.6)	260 (30.7)
3	328 (31.2)	256 (30.2)
Psychological factors (0–16)^a^	3 (1–5)	3 (1–5)
**Social class**		
High	170 (19.2)	157 (19.7)
Middle	378 (42.6)	344 (43.2)
Low	216 (24.4)	184 (23.1)
Unclassified	123 (13.9)	111 (13.9)
BMI, median (interquartile range)	17.8 (16.5–19.2)	17.7 (16.4–19.1)
Waist-to-height ratio, median (interquartile range)	0.43 (0.41–0.45)	0.43 (0.41–0.46)

^a^Measured at follow-up (2012).

﻿Over the course of 2 years, 40 children became overweight (two of which became obese), and only two of these had never experienced spinal pain at baseline (Table 2). The 2-year incidence rate of overweight was 5.29% (95%CI 3.98–7.58%) for children with spinal pain at baseline and 1.55% (95%CI 0.19–5.45%) for those without spinal pain at baseline, but the difference was not statistically significant. We saw a statistically significant trend with stepwise increases in the 2-year incidence of overweight for both increasing frequency of spinal pain (from 1.6 to 7.8%) and increasing number of pain sites (from 1.5 to 6.6%).

**Table 2:** The relationship between spinal pain at age 11–13 and overweight at age 13–15 in a school-based cohort with 848 normal-weight Danes (2010–2012) reporting both the theory driven and the data-driven models.

Pain status at baseline (11–13 yoa.)	Overweight^a^ at follow-up (13–15 yoa.)								
	n	2-year incidence rate	95% CI	Crude RR	95% CI	Adjusted RR^b^	95% CI	Adjusted RR^c^	95% CI
**Lifetime prevalence spinal pain**									
No (n = 129)	2	1.55%	0.19,5.45	1		1		1	
Yes (n = 719)	38	5.29%	3.98,7.58	3.41	0.83,13.96	3.60	0.87,14.96	3.55	0.85,14.68
**Frequency of spinal pain**									
Never (n = 129)	2	1.55%	0.19,5.45	1		1		1	
Once or twice (n = 337)	15	4.45%	2.51,7.24	2.87	0.67,12.38	3.23	0.75,14.01	3.06	0.71,13.24
Sometimes (n = 280)	15	5.36%	3.03,8.68	3.46	0.80,14.89	3.74	0.85,16.48	3.73	0.85,16.24
Often (n = 102)	8	7.84%	3.45,14.87	**5.06**	**1.10,23.31**	**4.95**	**1.02,24.07**	**5.36**	**1.13,25,41**
*P*	–	**0.025**^d^		**0.026**^**e**^		**0.049**^**e**^		**0.027**^**e**^	
**Number of spinal pain sites**									
0 (n = 127)	2	1.55%	0.19,5.45	1		1		1	
1 (n = 194)	9	4.43%	2.05,8.25	2.86	0.63,13.03	3.26	0.71,14.92	3.07	0.67,13.97
2 (n = 248)	12	4.62%	2.41,7.92	2.98	0.68,13.10	3.32	0.75,14.78	3.16	0.71,14.07
3 (n = 239)	17	6.64%	3.92,10.42	**4.28**	**1.00,18.26**	4.30	0.97,19.61	**4.50**	**1.04,19.54**
*P*	–	**0.034**^d^		**0.037**^**e**^		0.064^**e**^		**0.039**^**e**^	

Bold font indicates statistical significance.

*CI* confidence intervals, *RR* relative risk.

^a^Overweight is calculated by age and gender specific cut-points of Body Mass Index (BMI).

^b^Theory-driven model adjusted for sex, age, social class and psychological health, n = 796.

^c^Data-driven model adjusted for sex, psychological health and diet, n = 843.

^d^Based on an extension of the Wilcoxon rank-sum test as defined by Cuzick.

^e^Overall p-value for the association.

The crude regression analyses showed that the lifetime prevalence of spinal pain was strongly associated with later overweight (RR 3.41; 95% CI 0.83,13.96). Furthermore, a clear stepwise increase in risk for overweight with increasing frequency of pain at baseline (RR 5.06; 95% CI 1.10–23.31 for ‘often’ compared to ‘never’) and with increasing number of pain sites (RR 4.28; 95% CI 1.00–18.26 for three sites compared to none) was noted. The overall relative risk was statistically significant for the association with both frequency of spinal pain (p = 0.026) and with number of pain sites (p = 0.037). All estimates are presented in Table 2.

The theory-driven model which included sex, age, social class, and psychological factors demonstrated similar results with a stepwise increase in risk for overweight with increasing frequency of pain at baseline (adjusted RR 4.95; 95% CI 1.02–24.07 for ‘often’ compared to ‘never’) and with increasing number of pain sites (adjusted RR 4.30; 95% CI 0.97–19.61 for three sites compared to none). The overall relative risk was statistically significant for the association with frequency of spinal pain but not for number of pain sites in the fully adjusted model. All estimates are presented in Table 2.

In the data-driven model, bivariable analyses showed sex, psychological factors and diet to be associated with either SP at baseline and overweight at follow-up, and they were included in the model. None of them were related to both outcome and predictor, and therefore an approach including only apparently true confounders would be similar to the crude analyses.

Results from the bivariable analyses are shown in Appendix 1, Tables 3–6 (Supplementary Material). The data driven models also showed a stepwise increase with increasing frequency of pain at baseline (adjusted RR = 5.56;95% CI 1.17–26.43 for ‘often SP’ compared to ‘never’) and with increasing number of pain sites (adjusted RR = 4.59; 95% CI 1.05–19.98 for three pain sites compared to none).

The inherent risk of overadjustment by including variables only related to either predictor or outcome appeared to be negligible. All estimates of the data-driven model are also presented in Table 2.

### Sensitivity analysis

In the sensitivity analysis using waist-to-height ratio to classify normal- or overweight, 861 children were available for analysis of whom 56 were categorized as overweight at follow-up. No consistent patterns of associations with baseline spinal pain were detected. Results are shown in Appendix 2, Table 7 (Supplementary Material).

## Discussion

Although only 40 children developed overweight over the 2 years follow-up, a clear pattern emerged where children with more frequent and more widespread spinal pain had an increased risk of being overweight 2 years later.

Self-reported pain is by nature subjective, and thus can be heavily influenced by other factors; in this case most importantly psychological health as this is also a strong risk factor for the development of obesity^7^. However, the adjusted associations indicate that there is an association between spinal pain and subsequent overweight, independent of psychological factors. Interestingly, the difference between adjusted and unadjusted relative risks decreases as spinal pain increases, indicating that confounding is stronger for the less severe cases of SP.

Since several studies have shown that spinal pain can lead to decreased physical activity^14^, which in turn might cause overweight^7,9,10^, it is plausible that the relationship between spinal pain and overweight could be due to a mediating effect of decreased physical activity. Furthermore, a recent study has demonstrated an association between chronic spinal pain in adults and dietary intake. However, the direction of this association is unknown due to a cross-sectional design, thus diet is also potentially either a causative or a mediating factor, and as such should be included in future studies^35^. Future studies should include a larger sample, include both potential confounders and mediators, and follow the children over longer time in order to perform a full mediation analysis to explore the complex causal pathways of the association. Unfortunately, the present study does not have the power to do so, but simply demonstrates the presence of the association.

A major strength of this study is the school-based design, resulting in a high response rate which is likely to be non-differential as both anthropometric measurements and questionnaires were administered during school hours. However, the population might not be representative for the general Danish population in the age group. The schools were mainly located in small towns because the municipalities were selected from criteria such as grade of urbanization and “move-ability” which were necessary inclusion criteria in the SPACE Study^18^. We did not find any literature about adolescent spinal pain in different geographical groups or in relation to grade of urbanization, so how this might have influenced our results remains unclear.

Another strength is the use of questionnaires which are developed for, and validated in, the relevant age group and cultural setting^21^, as well as standardized anthropometric measurements performed by trained research assistants^18^.

The results of this study should spur further investigations into this area. Low back pain is responsible for more years lived with disability globally than any other health condition^11^, and it is well known that adolescents with back pain have a high risk of backpain in adulthood^36^. Our results suggest that back pain may also predispose teenagers for later overweight and possibly obesity, which is another reason why a greater focus on primary and secondary prevention of spinal pain should start already during school age.

## Conclusion

In this cohort of Danish school children, a pattern emerged where more spinal pain (more frequent or in more sites) increased the risk of overweight 2 years later.

## Research in context

### Evidence before this study

We did a literature search in PubMed on August 30, 2020, for scientific papers published from database inception that examined the relationship between back or neck pain and overweight in children or adolescents. The search terms used were “back pain”, “neck pain” “child*”, “adolescence”, “adolescent”, “overweight”, and “obesity”, with no limits applied to publication dates or language. Further studies were identified from the reference lists of the preliminary search results. Most of the studies investigating associations between overweight and back or neck pain, were either cross-sectional or investigated the role of overweight as a risk factor for musculoskeletal disorders. Only one study, published in 2000, investigated the reverse association—back pain as a risk factor for overweight—and found that chronic back pain at age 23 increased the risk of subsequent weight gain.

Considering the rise in obesity, even at a young age, and the resulting increased morbidity and mortality, all modifiable risk factors should be explored. Back and neck pain are very prevalent in the young and thus an important target for intervention, if a temporal association can be established.

### Added value of this study

This study indicates a potential link between back or neck pain in early adolescence and subsequent overweight. In a nationally representative cohort of normal weight 11–13-year-old children, we found that back or neck pain at baseline increased the risk of overweight 2 years later. Furthermore, higher frequency of pain at baseline resulted in higher relative risks of overweight.

### Implications of all the available evidence

Our results indicate that back and neck pain may contribute to the development of obesity in adolescence, and therefore measures to improve spinal health in children and adolescents should be prioritized. Such measures might not only influence lifetime trajectories of spinal health but might also have the potential to influence the alarming rise in obesity.

## Supplementary Information

Supplementary Information 1.

Supplementary Information 2.

## Data Availability

Individual participant data that underlie the results reported in this article, after de-identification can be made available to researchers who provide a methodologically sound proposal. Proposals should be directed to l.hestbaek@nikkb.dk; to gain access, data requestors will need to sign a data access agreement.

## Abbreviations

BMI: Body mass index
CI: Confidence interval
RR: Relative risk
SPACE: School site, play spot, active transport, club fitness and Environment
