# Supplementary material for: Spinal pain increases the risk of becoming overweight in Danish schoolchildren
Source: Sci Rep. 2021 May 13;11:10235. doi: 10.1038/s41598-021-89595-5 (PMC8119474; doi:10.1038/s41598-021-89595-5)
Supplement: Supplementary file 1 — Supplementary Information 1. [file 41598_2021_89595_MOESM1_ESM.docx]

| Table 3 Associations between the potential confounders and overweight (BMI) in 848 normal-weighted Danes aged 11-13 yrs., 2010. | | | | | | |
| --- | --- | --- | --- | --- | --- | --- |
|  | | **Overweight status at follow-up (n=866)** | | | | p<0.10 |
|  |  | Yes (n=40)  (n) | Incidence rate | No (n=808)  (median, 95% CI) | Yes (n=40)  (median, 95% CI) |  |
| Sex | Boys (n=448) | 22 | 4.84 |  |  | No |
|  | Girls (n=400) | 18 | 4.38 |  |  |  |
| Age (yrs.) (n=848) | |  |  | 12.6 (12.5- 12.7) | 12.6 (12.3-12.9) | No |
| Psychological factors (n=848) | |  |  | 3 (2-3) | 3 (1-4) | No |
| Physical activity (counts per minute) (n=841) | |  |  | 566 (549-583) | 517 (445-593) | No |
| Social class (n=796) | High (n=160) | 6 | 3.75 |  |  | Yes |
|  | Middle (n=347) | 11 | 3.17 |  |  |  |
|  | Low (n=190) | 16 | 8.42 |  |  |  |
|  | Unclassified (n=114) | 5 | 4.39 |  |  |  |
| Diet (# of servings of fruit and/or vegetables per day) (n=845) | |  |  |  |  |  |
|  | <6 (n=453) | 13 | 2.74 |  |  | Yes |
|  | ≥6 (n=340) | 27 | 7.30 |  |  |  |

| Table 4 Associations between the potential confounders and lifetime prevalence of spinal pain in 848 normal-weighted Danes aged 11-13 yrs., 2010. | | | | | |
| --- | --- | --- | --- | --- | --- |
|  | | **Spinal pain status at baseline (n=848)** | | | p<0.10 |
|  |  | No (n=129)  n(%)/median,95%CI | Yes (n=719)  n(%)/median,95%CI |  |  |
| Sex (n=848) | Boys (n=448) | 79 (61) | 369 (51) |  | yes |
|  | Girls (n=400) | 50 (39) | 350 (49) |  |  |
| Age (yrs.) (n=848) | | 12.7 (12.4 – 12.8) | 12.6 (12.5 – 12.6) |  | no |
| Psychological factors (n=848) | | 1 (0-2) | 3 (3-3) |  | yes |
| Physical activity (counts per minute) (n=841) | | 584 (531 – 644) | 565 (548 – 582) |  | no |
| Social class (n=796) | High | 25 (20) | 132 (20) |  | no |
|  | Middle | 52 (41) | 292 (44) |  |  |
|  | Low | 33 (26) | 151 (23) |  |  |
|  | Unclassified | 17 (13) | 94 (14) |  |  |
| Diet (# of servings of fruit and/or vegetables per day) (n=843) | |  |  |  |  |
|  | <6 | 33 (26) | 173 (24) |  | no |
|  | ≥6 | 96 (74) | 541 (76) |  |  |

| Table 5 Associations between the potential confounders and frequency of spinal pain in 848 normal-weighted Danes aged 11-13 yrs., 2010. | | | | | | | |
| --- | --- | --- | --- | --- | --- | --- | --- |
|  | | **Frequency of spinal pain at baseline (n=848)** | | | | | p<0.10 |
|  |  | Never  (n=129)  n(%)/median,95%CI | Once or twice  (n=337)  n(%)/median,95%CI | Sometimes  (n=280)  n(%)/median,95%CI | Often  (n=102)  n(%)/median,95%CI |  |  |
| Sex (n=848) | Boys (n=448) | 79 (61) | 170 (50) | 131 (47) | 49 (48) |  | no |
|  | Girls (n=400) | 50 (39) | 167 (50) | 149 (53) | 53 (52) |  |  |
| Age (yrs.) (n=848) | | 12.7 (12.4-12.9) | 12.5 (12.3-12.6) | 12.7 (12.6-12.8) | 12.6 (12.3-12.8) |  | no |
| Psychological factors (n=848) | | 1(0-2) | 2 (2-3) | 3 (3-4) | 5 (4-6) |  | yes |
|  |  |  |  |  |  |  |  |
| Physical activity (counts per minute) (n=841) | | 584 (531-643) | 560 (541-587) | 566 (529-591) | 584 (543-646) |  | no |
|  | |  |  |  |  |  |  |
| Social class (n=796) | High (n=157) | 25 (20) | 56 (18) | 53 (20) | 23 (24) |  | no |
|  | Middle (n=344) | 134 (41) | 134 (43) | 124 (47) | 34 (36) |  |  |
|  | Low (n=184) | 69 (22) | 68 (22) | 59 (23) | 24 (25) |  |  |
|  | Unclassified (n=111) | 54 (17) | 54 (17) | 26 (10) | 14 (15) |  |  |
| Diet (# of servings of fruit and/or vegetables per day) (n=843) | |  |  |  |  |  |  |
|  | <6 (n=129) | 33 (26) | 84 (25) | 70 (25) | 19 (19) |  | no |
|  | ≥6 (n=714) | 98 (74) | 250 (75) | 209 (75) | 82 (81) |  |  |

| Table 6 Associations between the potential confounders and number of spinal pain sites in 848 normal-weighted Danes aged 11-13 yrs., 2010. | | | | | | | |
| --- | --- | --- | --- | --- | --- | --- | --- |
|  | | **Number of spinal pain sites at baseline (n=848)** | | | | | p<0.10 |
|  |  | 0  (n=132)  n(%) or  median,95%CI | 1  (n=200)  n(%) or  median,95%CI | 2  (n=262)  n(%) or  median,95%CI | 3  (n=254)  n(%) or  median,95%CI |  |  |
| Sex (n=848) | Boys (n=448) | 79 (61) | 105 (52) | 133 (51) | 131 (51) |  | no |
|  | Girls (n=400) | 50 (38) | 98 (48) | 127 (49) | 125 (49) |  |  |
| Age (yrs.) (n=848) | | 12.7 (12.4-12.9) | 12.6 (12.4-12.6) | 12.6 (12.4-12.7) | 12.6 (12.5-12.7) |  | no |
| Psychological factors (n=848) | | 1 (0-2) | 2 (2-2) | 3 (3-4) | 4 (3-5) |  | yes |
| Physical activity (counts per minute) (n=841) | | 583 (531-644) | 562 (528-588) | 570 (538-600) | 562 (539-590) |  | no |
| Social class (n=796) | High (n=157) | 25 (20) | 42 (22) | 37 (16) | 53 (22) |  | no |
|  | Middle (n=344) | 52 (41) | 91 (47) | 96 (40) | 105 (44) |  |  |
|  | Low (n=184) | 33 (26) | 35 (18) | 68 (29) | 48 (20) |  |  |
|  | Unclassified (n=111) | 17 (13) | 25 (13) | 37 (16) | 32 (13) |  |  |
| Diet (# of servings of fruit and/or vegetables per day) (n=843) | |  |  |  |  |  |  |
|  | <6 (n=206) | 32 (26) | 58 (29) | 61 (24) | 54 (21) |  | no |
|  | ≥6 (n=637) | 96 (74) | 144 (71) | 197 (76) | 200 (79) |  |  |
