# Supplementary material for: Spinal pain increases the risk of becoming overweight in Danish schoolchildren
Source: Sci Rep. 2021 May 13;11:10235. doi: 10.1038/s41598-021-89595-5 (PMC8119474; doi:10.1038/s41598-021-89595-5)
Supplement: Supplementary file 2 — Supplementary Information 2. [file 41598_2021_89595_MOESM2_ESM.docx]

| Table 7, Sensitivity analysis: The Association Between Spinal Pain at age 11-13 And Overweight Defined by Waist-To-Height Ratio at age 13-15 in a School-Based Cohort of Normal-Weighted Danes (2010-12) | | | | | | |
| --- | --- | --- | --- | --- | --- | --- |
| Pain status at baseline  (11-13 yoa.) | | Overweight^†^ status at follow-up (13-15 yoa.) | | | | |
|  |  | Over-weight, n  (n=56) | 2-year incidence rate (95%CI) | | Minimally adjusted RR ^a^  (95% CI) | Fully adjusted RR ^b^  (95% CI) |
| **Lifetime prevalence spinal pain (n=861)** | |  |  | |  |  |
| No (n=130) | | 8 | 6.15% (2.69%-11.77%) | | 1 | 1 |
| Yes (n=731) | | 48 | 6.57% (4.88%-8.61%) | | 1.07 (0.52 – 2.20) | 0.89 (0.42-1.86) |
| **Frequency of spinal pain (n=861)** |  | | |  |  |  |
| Never (n=130) | | 8 | 6.15% (2.69%-11.77%) | | 1 | 1 |
| Once or twice (n=344) | | 20 | 5.81% (3.59%-8.84%) | | 0.87 (0.39 – 1.90) | 0.73 (0.32-1.67) |
| Sometimes (n=284) | | 18 | 6.39% (3.80%-9.83%) | | 0.96 (0.43 – 2.15) | 0.92 (0.40-2.11) |
| Often (n=103) | | 10 | 9.71% (4.75%-17.13%) | | 1.43 (0.59 – 3.48) | 1.51 (0.60-3.80) |
| p | |  |  | | 0.362 ^d^ | 0.258 ^d^ |
| **Number of spinal pain sites (n=861)** | |  |  | |  |  |
| 0 (n=130) | | 8 | 6.15% (2.69%-11.77%) | | 1 | 1 |
| 1 (n=206) | | 11 | 5.34% (2.70%-9.35%) | | 0.81 (0.34 – 1.95) | 0.61 (0.24-1.60) |
| 2 (n=265) | | 23 | 8.68% (5.58%-12.74%) | | 1.28 (0.59 – 2.77) | 1.20 (0.55-2.65) |
| 3 (n=260) | | 14 | 5.38% (2.97%-8.87%) | | 0.82 (0.35 – 1.89) | 0.78 (0.33-1.87) |
| p | |  |  | | 0.925 ^c^ | 0.974 ^c^ |
| Abbreviations: CI= Confidence intervals; RR= Relative risk  ^†^ Overweight is calculated height-to-waist ratio ≥0.5  ^a^ Adjusted for age and sex  ^b^ Adjusted for sex, age, social class and psychological factors  ^c^ Overall p-value for the association | | | | | | |
